# *DNMT3A*^R882^-associated hypomethylation patterns are maintained in primary AML xenografts, but not in the *DNMT3A*^R882C^ OCI-AML3 leukemia cell line

**DOI:** 10.1038/s41408-018-0072-9

**Published:** 2018-04-04

**Authors:** David Chen, Matthew Christopher, Nichole M. Helton, Ian Ferguson, Timothy J. Ley, David H. Spencer

**Affiliations:** 1 Division of Dermatology, and Section of Stem Cell Biology, Department of Internal Medicine, Washington University School of Medicine, St. Louis, MO USA; 2 Division of Oncology, Section of Stem Cell Biology, Department of Internal Medicine, Washington University School of Medicine, St. Louis, MO USA; 3 McDonnell Genome Institute, Washington University, St. Louis, MO USA

*DNMT3A*^R882^ mutations act as dominant negative alleles in vitro^1^ and are associated with focal regions of DNA hypomethylation in primary acute myeloid leukemia (AML) samples and non-leukemic hematopoietic cells^2^. In primary AML cells, this hypomethylation manifests both as methylation loss and attenuated CpG island hypermethylation relative to normal hematopoietic stem/progenitor cells. Although *DNMT3A*^R882^ mutations have a clear effect on DNA methylation in AML cells, the functional consequences of these changes are not yet clear. Future study of the downstream effects of mutant *DNMT3A*-associated hypomethylation will require model systems to investigate the genomic targets that are affected, and to understand whether these changes alter gene regulation in ways that promote leukemogenesis. Examples of model systems include genetically modified mice, patient-derived xenografts, and human cell lines containing *DNMT3A*^R882^ mutations. The methylation phenotypes of mice lacking Dnmt3a, or expressing mutant *Dnmt3a* alleles, have been reported previously^3–6^, but much less is known about whether alterations in methylation caused by *DNMT3A*^R882^ alleles are retained in either patient-derived xenografts or human AML cell lines, and whether these models could therefore be used to accurately represent *DNMT3A*^R882^-dependent methylation changes in AML cells.

To address this question, we performed whole-genome bisulfite sequencing (WGBS) using DNA from OCI-AML3 cells, which is the only leukemia cell line currently known to have a native *DNMT3A*^R882C^ mutation^7^. We also evaluated four xenografts derived from a primary AML sample containing the *DNMT3A*^R882H^ mutation. The OCI-AML3 line was obtained from the DSMZ cell collection and cultured via recommended conditions before DNA extraction at two independent passages for WGBS. The presence of the *DNMT3A*^R882C^ allele in these cells was verified via targeted sequencing prior to methylation analysis (Supplementary Figure S1), as were the recurrent *NPM1* exon 12 insertion (NPMc) and the *NRAS*^Q61L^ mutation. No functional mutations were identified in other recurrently mutated AML genes with roles in epigenetic modification, such as *IDH1*, *IDH2*, *ASXL1*, *EZH2*, or *TET2*. Two missense variants of unknown significance were present in *TET1* (Supplementary Table S2), which is not frequently mutated in AML samples. Importantly, we saw no evidence for amplification of the wild-type *DNMT3A* allele in this cell line (data not shown). We also extracted two replicate DNA samples from comparator AML cell lines that are wild-type for *DNMT3A*, including Kasumi-1 and NB4, which have a t(8;21) translocation (creating the *RUNX1-RUNX1T1* fusion gene) and a t(15;17) translocation (resulting in a *PML*-*RARA* fusion), respectively. Patient-derived AML xenografts were generated in two independent humanized NSG mice (NSG-SGM3) from a primary AML sample with the *DNMT3A*^R882H^ mutation (along with *NPM1* and *FLT3*-ITD mutations; AML 721214, described as AML88 in ref. ^8^; Supplementary Table S1) via tail vein injection of 1 million cells. Mice were killed at 16 weeks and flow cytometry analysis of bone marrow confirmed high human AML cell engraftment (90% and 81% human CD45 chimerism in the marrow, respectively; Supplementary Figure S2). Engrafted cells were subsequently transferred for two additional passages in multiple mice, and DNA was isolated from unmanipulated, xenografted bone marrow cells from both primary and tertiary passages in duplicate. WGBS libraries were prepared from all samples (including the primary AML sample used for xenotransplantation) with 50ng of DNA using the Swift DNA methylation library prep kit and sequenced on Illumina HiSeq X instruments to obtain 277 million to 1.5 billion paired 150 bp reads per sample, which yielded a median of 4 to 50-fold coverage for at least 26 million CpGs in the human reference genome for each sample (Supplementary Table S1).

We first performed comparisons of genome-wide DNA methylation levels between the AML cell lines, data from normal human CD34^+^ cells, and primary AML samples with and without *DNMT3A*^R882^ mutations^2^. The primary AML samples demonstrated methylation patterns that were previously reported to be associated with *DNMT3A*^R882^ mutant AML samples, including lower methylation overall, and at CpG island-shores, compared to normal CD34 + cells. We also detected attenuated hypermethylation at CpG islands compared to AMLs with wild-type *DNMT3A* (Fig. 1a). In distinct contrast, CpGs in all three AML cell lines were strikingly hypermethylated at CpG islands and island-shores relative to the primary human cell samples (34–51% mean methylation in the cell lines at CpG islands, vs. 17–19% in the AML samples), which is consistent with previous studies of methylation in cancer cell lines compared to normal tissues^9–11^. Interestingly, the mean methylation of OCI-AML3 and NB4 cells across the entire genome was dramatically lower than all other samples (68 and 64% for these two cell lines, vs. 85% mean methylation for all other samples; see Fig. 1a). This difference was manifest primarily as an increase in large “partially-methylated domains” (PMDs; Supplementary Figure S3), a phenomenon that has been observed previously in some cell lines regardless of *DNMT3A* mutation status, and that is associated with transcriptionally inactive genomic regions^12^. The number of PMDs was similar between the OCI-AML3 and NB4 cell lines (Figure S3A and S3B), indicating that these features in OCI-AML3 cells cannot be uniquely attributed to the *DNMT3A*^R882^ mutation. In contrast to all three cell lines, methylation levels in the patient-derived xenografts from AMLs with *DNMT3A*^R882H^ closely resembled primary AML cells from the same tumor (and other AML samples with *DNMT3A*^R882H^ mutations) in all genomic regions, including subtle hypomethylation at CpG island-shores, and attenuated hypermethylation of CpG islands (Fig. 1a, blue points), as we described previously^2^.

**Fig. 1:**
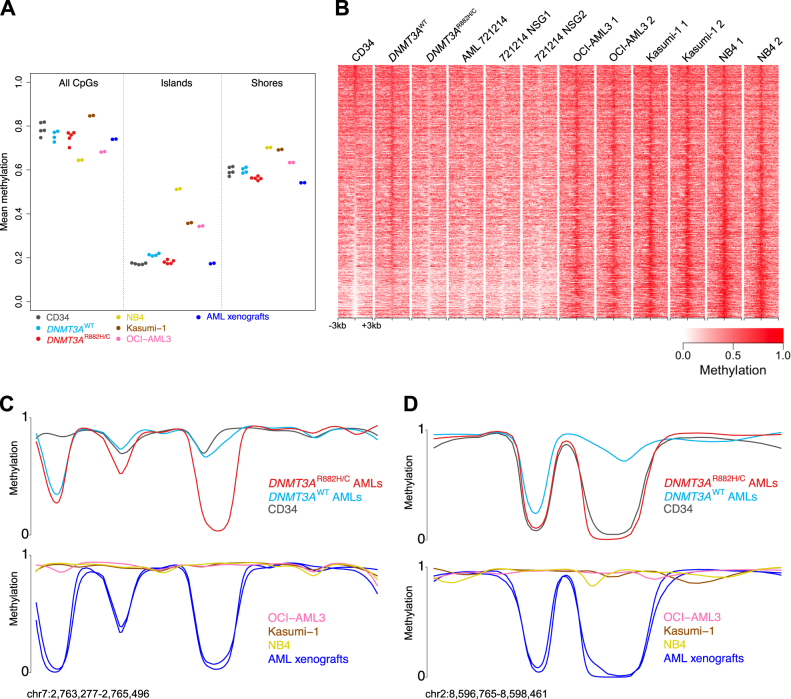
*DNMT3A*^R882^-associated methylation patterns in primary AML cells, patient-derived xenografts, and AML cell lines. **a** Genome-wide methylation statistics for all samples. Points show mean methylation values for all CpGs, CpG islands, and CpGs in island-shores for each sample. Note that the primary AML samples with *DNMT3A*^R882^ mutations include four previously published samples^2^, and a fifth sample that was used to generate AML xenografts in four independent mice. **b** Methylation patterns at 3898 DNMT3A-dependent differentially methylated regions (DMRs) from primary AML samples, AML cell lines, and xenografts. Each heatmap shows the mean methylation in 50 bp windows for a 6 kb window centered on a DMR locus (one DMR per row), with methylation represented on a white (unmethylated) to red (methylated) scale. **c**, **d** Example DMRs with either *DNMT3A*^R882^-associated hypomethylation (**c**), or loci with DNMT3A-mediated hypermethylation that is absent in AMLs with *DNMT3A*^R882^ (**d**). The top tracks in each panel show mean methylation from normal CD34 cells (*N* = 5), AML samples with and without *DNMT3A*^R882H/C^ (*N* = 4 each), and the bottom tracks show the mean methylation from OCI-AML3, NB4, and Kasumi-1 cells (*N* = 2, each), and individual methylation levels for two AML xenografts derived from AML sample 721214. All the methylation data in these panels were smoothed^16^ prior to plotting. Coordinates refer to human reference sequence build 37 (hg19)

We next used results from our previously published study of DNMT3A-dependent methylation in AML to determine whether the 3,898 differentially methylated regions (DMRs) that were hypomethylated in primary AML cells with *DNMT3A*^R882^ were maintained in the OCI-AML3 cells and patient xenografts. The OCI-AML3 genome was not hypomethylated at these loci, but in fact was hypermethylated relative to both AMLs with *DNMT3A*^R882^ and normal CD34 cells (Fig. 1b). Statistical analysis of these regions demonstrated that 81% (3,183/3,898) of the DMRs were hypermethylated in OCI-AML3 cells compared to normal CD34 cells (Supplementary Figure S4A), and 90% (3517/3898) were hypermethylated compared to the primary *DNMT3A*^R882^ AML samples (Supplementary Figure S4B); a similar number of DMRs were hypermethylated in Kasumi-1 and NB4 cells (85 and 82% vs. CD34 cells; 91 and 95% vs. *DNMT3A*^R882^ AML samples, respectively; Supplementary Figures S4C-F). We have shown that hypomethylation in primary AML samples with *DNMT3A*^R882^ reflects both methylation loss, and reduced CpG island hypermethylation relative to normal CD34 cells^2^; a review of individual DMR loci from both of these categories demonstrates that OCI-AML3 cells failed to recapitulate either of these phenotypes (Fig. 1c, d). We performed the same analysis on the data from the primary AML sample with the *DNMT3A*^R882^ mutation that was used for xenotransplantation, and the two passaged tumor cell populations from this sample: all three were hypomethylated relative to the AML samples that were wild-type for *DNMT3A* at most DMRs (e.g., > 73% of DMRs were statistically hypomethylated relative to *DNMT3A*^WT^ AML samples, Supplementary Figures S4E-G). Xenotransplanted cells remained hypomethylated at these loci following two additional passages through NSG-SGM3 mice (Supplementary Figure S5), and the methylation relationships between samples with *DNMT3A*^R882^ and normal CD34 cells were also preserved in all transplanted AML cells (Fig. 1c, d, and Supplementary Figure S4).

Given the virtual absence of the focal, canonical hypomethylation phenotype in the OCI-AML3 cell line, we performed additional experiments to assess the function of *DNMT3A*^*R882*^ in this cell line. We verified that the mutant and wild-type alleles of *DNMT3A* were expressed equally in two replicate RNA-seq experiments (Supplementary Figure S6A). Overall expression levels of both *DNMT3A* and *DNMT3B* (including active and inactive isoforms) and other genes involved in DNA methylation were also similar between OCI-AML3 cells and a previously published set of 32 primary AML samples^2^, although expression of *DNMT1* and *BCAT1*^13^ were substantially higher in OCI-AML3 cells (Supplementary Figure S6B). Surprisingly, the bulk in vitro methylation activity of OCI-AML3 cell lysates performed on an unmethylated DNA substrate^1^ was significantly higher than Kasumi-1 cell lines (Figure S6C), even though Kasumi-1 cells have significantly higher CpG methylation across the genome, suggesting that de novo methylation in these cells is probably influenced by factors other than *DNMT3A*^R882^.

Models of *DNMT3A*^R882^ that accurately recapitulate the epigenetic phenotype of primary AML samples with this mutation will be critical to understand its functional consequences, and investigate targeted therapies. In this study, we found that *DNMT3A*^R882^-associated hypomethylation was preserved in patient-derived AML xenografts with *DNMT3A*^R882^, which displayed the same global and focal hypomethylation phenotypes as primary patient samples. The OCI-AML3 cell line, which harbors a *DNMT3A*^R882C^ allele, showed none of these patterns, and were in fact hypermethylated at many of the DNMT3A-dependent loci. Although these cells have been used to represent AML samples with *DNMT3A*^R882^ mutations^5,14,15^, they are clearly not an appropriate model for understanding *DNMT3A*^R882^-dependent methylation phenotypes in AML cells, or for making inferences about specific genes or loci that may be dysregulated by *DNMT3A*^R882^. We have proposed that CpG island hypermethylation may be a normal response to abnormal proliferation in leukemic cells; these data suggest that the residual de novo methylation activity present in OCI-AML3 cells is adequate to methylate these DNMT3A-dependent regions during long periods of cell culture. It is also possible that these cells never possessed the *DNMT3A*^R882^ methylation signature, although previous analysis has shown that primary AML samples with *DNMT3A*^R882^ invariably display some level of focal hypomethylation at the loci examined here. Moreover, the similarities between OCI-AML3 and other AML cells lines with different initiating mutations suggests that the methylation patterns in these cells may be related to properties that are associated with immortalization. Regardless, the methylation patterns in OCI-AML3, cells are clearly very different from primary AML samples with *DNMT3A*^R882^ mutations, and therefore this cell line is not an appropriate model for understanding genomic patterns of DNA methylation that are caused by the *DNMT3A*^R882^ mutation.

## Electronic supplementary material

Supplemental Figure Legends

Supplemental Figures

Supplemental Table S1

Supplemental Table S2
