# Supplementary material for: DNMT3AR882-associated hypomethylation patterns are maintained in primary AML xenografts, but not in the DNMT3AR882C OCI-AML3 leukemia cell line
Source: Blood Cancer J. 2018 Apr 4;8(4):38. doi: 10.1038/s41408-018-0072-9 (PMC5884841; doi:10.1038/s41408-018-0072-9)
Supplement: Supplementary file 1 — Supplemental Figure Legends [file 41408_2018_72_MOESM1_ESM.docx]

**Figure S1. Confirmation of *DNMT3A*^R882C^ in the OCI-AML3 cell line.** Targeted next-generation sequencing was performed on DNA extracted from OCI-AML3 cells using a previously described (TCGA NEJM 2012) 264 gene panel that includes all exons of *DNMT3A*. Manual review of the sequencing reads as shown confirmed the presence of a G to A transition at position 2:25,457,243 (build 37 coordinates).

**Figure S2. Bone marrow chimerism of NSG-SGM3 mice engrafted with primary human AML sample 721214 (AML88).** Bone marrow and spleen cells were harvested from two mice engrafted with AML 7217214 and stained for human (CD45-PerCp-Cy5.5, clone 2D1, eBioscience) and mouse CD45. Panels A and B show flow cytometric analysis of these data, and demonstrate that the bone marrow cells of these mice were comprised of 90% and 81% positive for the human CD45 cell surface marker.

**Figure S3. Accentuated partially-methylated domains in OCI-AML3 cells.** A) Low-resolution views of WGBS data from the Kasumi-1, NB4, and OCI-AML3 cell lines (two replicates each), along with two *DNMT3A*^WT^ and *DNMT3A*^R882H^ primary AML samples for chromosome 3. The chromosome is punctuated with numerous regions of low methylation (mean levels ~0.5) in NB4 and OCI-AML3 cells (blue and green tracks), compared to Kasumi-1 cells, which are nearly completely methylated across these regions (in red); both sets of AML samples also show consistently high methylation across the genome at this resolution. Panel B shows a barplot of the number of partially methylated domains across the entire genome, defined by the pmd program in the methpipe methylation analysis software suite (Song Q Plos One 2013), using the non-default parameter ‘-b 100000’.

**Figure S4. Methylation levels at *DNMT3A*^R882^-associated differentially methylated regions (DMRs) in leukemia cell lines and AML xenografts vs. primary CD34 cells and AML samples.** Panels show scatter plots of methylation for 3,898 DMRs identified in Reference 2. Each point shows the methylation level on a 0 to 1 scale for a single DMR in the samples indicated on the X and Y axes. Panels A-F show methylation at these loci in OCI-AML3, Kasumi-1, and NB4 cells (mean of 2 replicates each) vs. either normal CD34 cells (mean of 5 samples) or AMLs with a *DNMT3A*^R882^ mutation (mean of 4 samples). Panel G shows methylation in AML 721214 vs. AML samples with wild-type *DNMT3A* (mean of 4 samples). Panels H and I show individual xenografts generated from AML 721214 vs. the same set of 4 *DNMT3A*^WT^ AMLs. In all panels, red points indicate DMRs that are statistically significantly different between the samples (P<0.01, chi-squared test of methylated and unmethylated counts for each sample or summed over the sample group).

**Figure S5. Methylation at *DNMT3A*-dependent DMRs in primary AML samples and xenografts.** Locus heatmaps showing DNA methylation from primary human cells and mouse xenografts at 3,898 DMRs (each row is one DMR +/-3 kbp) previously shown to be dependent on functional DNMT3A (Spencer et al., Cell 2017). From the left to right, the heatmaps show mean DNA methylation in these regions from primary human CD34 cells (N=5), primary AML samples with and without a *DNMT3A*^R882^ mutation (N=4 each), and mouse xenografts after one (NSG1 and NSG2) or three (tertiary 1 and tertiary 3) passages (N=1 each). The DMRs are identical to those shown in Figure 1, and the same primary AML sample (UPN 721214/AML88) was use for all xenografts. Comparison of primary AML samples and all xenografts demonstrates that *DNMT3A*^R882^-associated methylation changes are retained in xenografted cells after primary and tertiary transplants.

**Figure S6. Expression of DNA methylation-associated genes in AML cell lines.** A) Allele counts of mutant and wild-type alleles of *DNMT3A*^R882C^ from RNA-seq data. Shown are the raw read alignments from RNA-seq of OCI-AML3 cells, which demonstrate that both the mutant (A) and wild-type (G) alleles are expressed equally in OCI-AML3 cells. B) Expression levels of DNA methyltransferase genes *DNMT3A*, *DNMT3B*, and *DNMT1*, as well as a selection of genes encoding proteins related to DNA methylation in 32 primary AML samples (from Reference 2) and OCI-AML3, NB4, and Kasumi-1 cell lines from RNA-sequencing. Expression levels are shown in transcripts per million (TPM) and are based on RNA-seq data following read trimming (with cutadapt [Marcel Martin EMBnet.journal 2017]), aligned with STAR (Dobin et al., Bioinformatics 2013), and transcript abundance estimation with stringtie (Pertea M et al., Nature Biotechnology 2015). Active vs. inactive *DNMT3B* isoforms were determined as in Reference 1, and values represent TPM levels summed over all transcripts in each group. C) *In vitro* methylation activity of cell lysates from OCI-AML3 and Kasumi-1 cells. Bulk de novo DNA methylation activity was assessed using whole cell lysates from OCI-AML3 and Kasumi-1 cells (N=8 each) as described in Reference 1, and is represented in counts per minute (CPM). Each point on the figure represents one experimental replicate. Median values for OCI-AML3 and Kasumi-1 cells were 1,137 and 613 CPM, respectively, which were statistically significantly different (P=0.0002, two-tailed Mann-Whitney U test). Background activity was obtained from a reaction without any cell lysate is also shown (in black).
