# Supplementary material for: DNMT3AR882-associated hypomethylation patterns are maintained in primary AML xenografts, but not in the DNMT3AR882C OCI-AML3 leukemia cell line
Source: Blood Cancer J. 2018 Apr 4;8(4):38. doi: 10.1038/s41408-018-0072-9 (PMC5884841; doi:10.1038/s41408-018-0072-9)

Figure S1. Confirmation of *DNMT3A*^{R882C} in the OCI-AML3 cells by targeted sequencing.

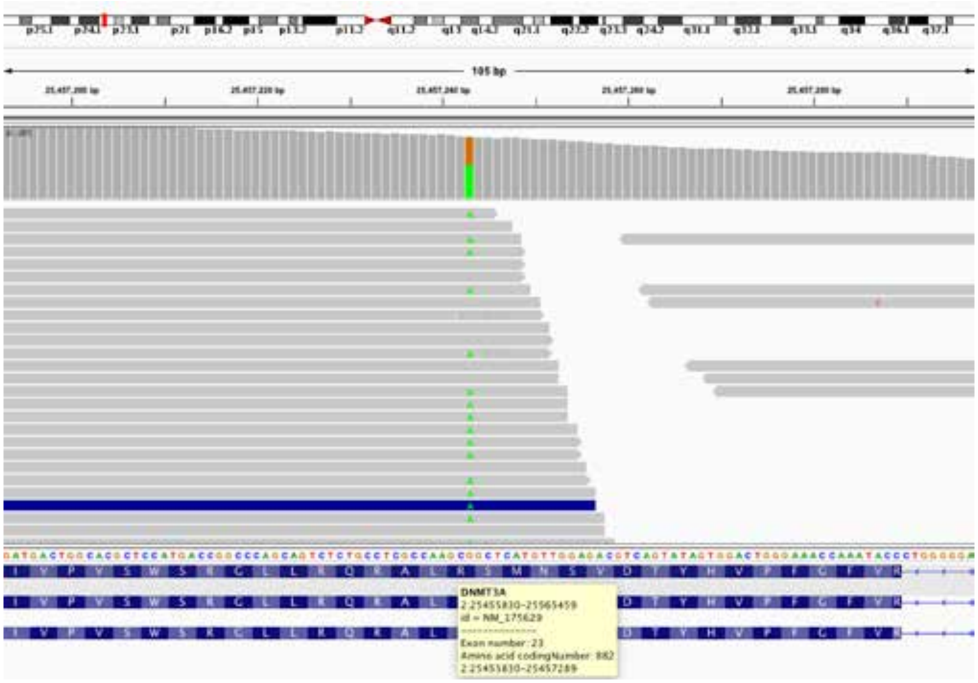

Figure S2. Chimerism of mouse xenografts generated using human AML 721214

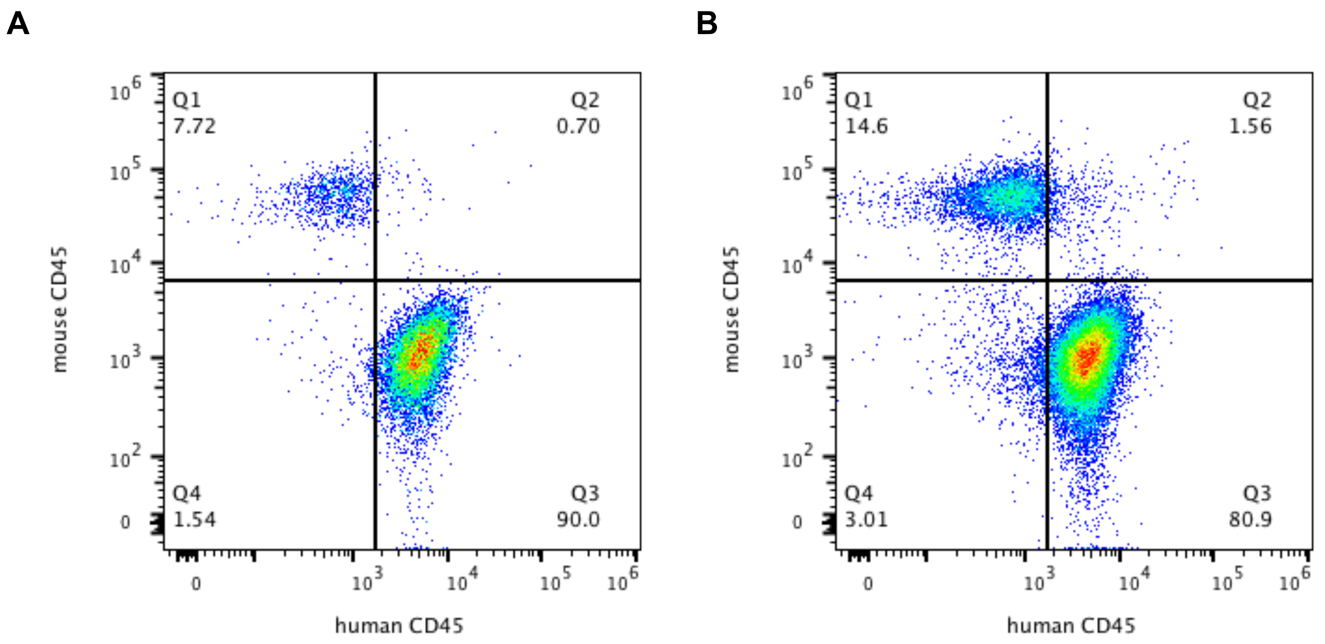

Figure S3. Partially methylated domains in OCI-AML3 cells vs. other cell lines

A

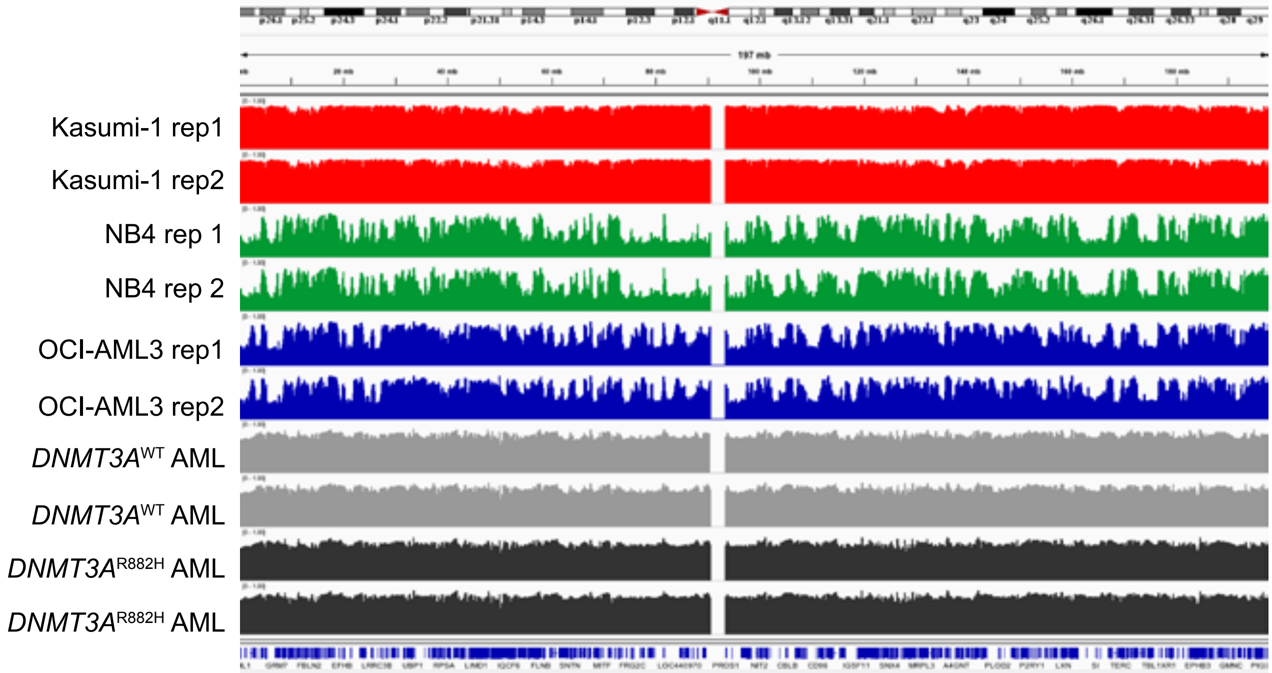

B

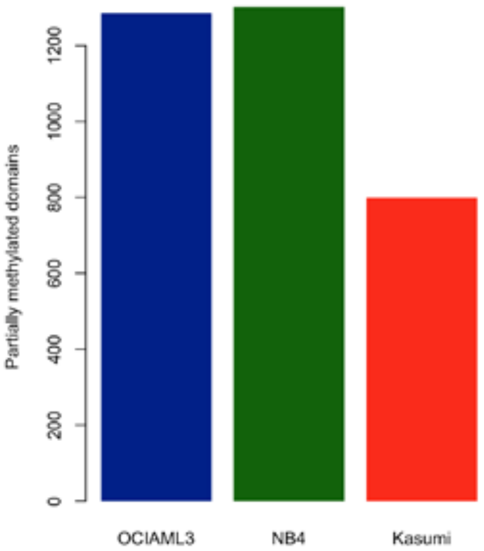

Figure S4. Methylation at 3,898 DNMT3A-dependent DMRs in cell lines and AML xenografts

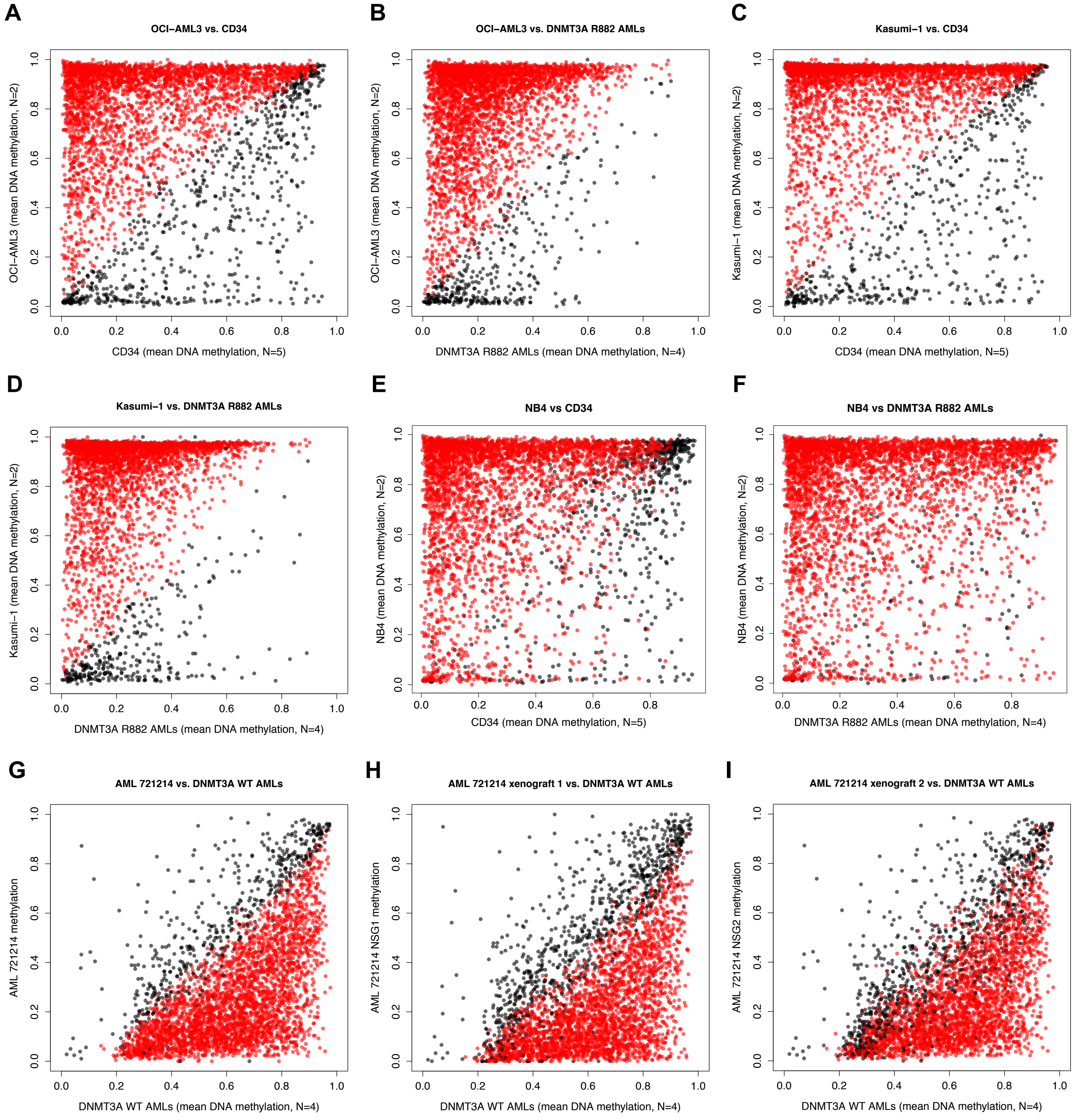

Figure S5. *DNMT3A*^{R882}-associated DMRs in primary AML samples, and primary and tertiary mouse xenografts

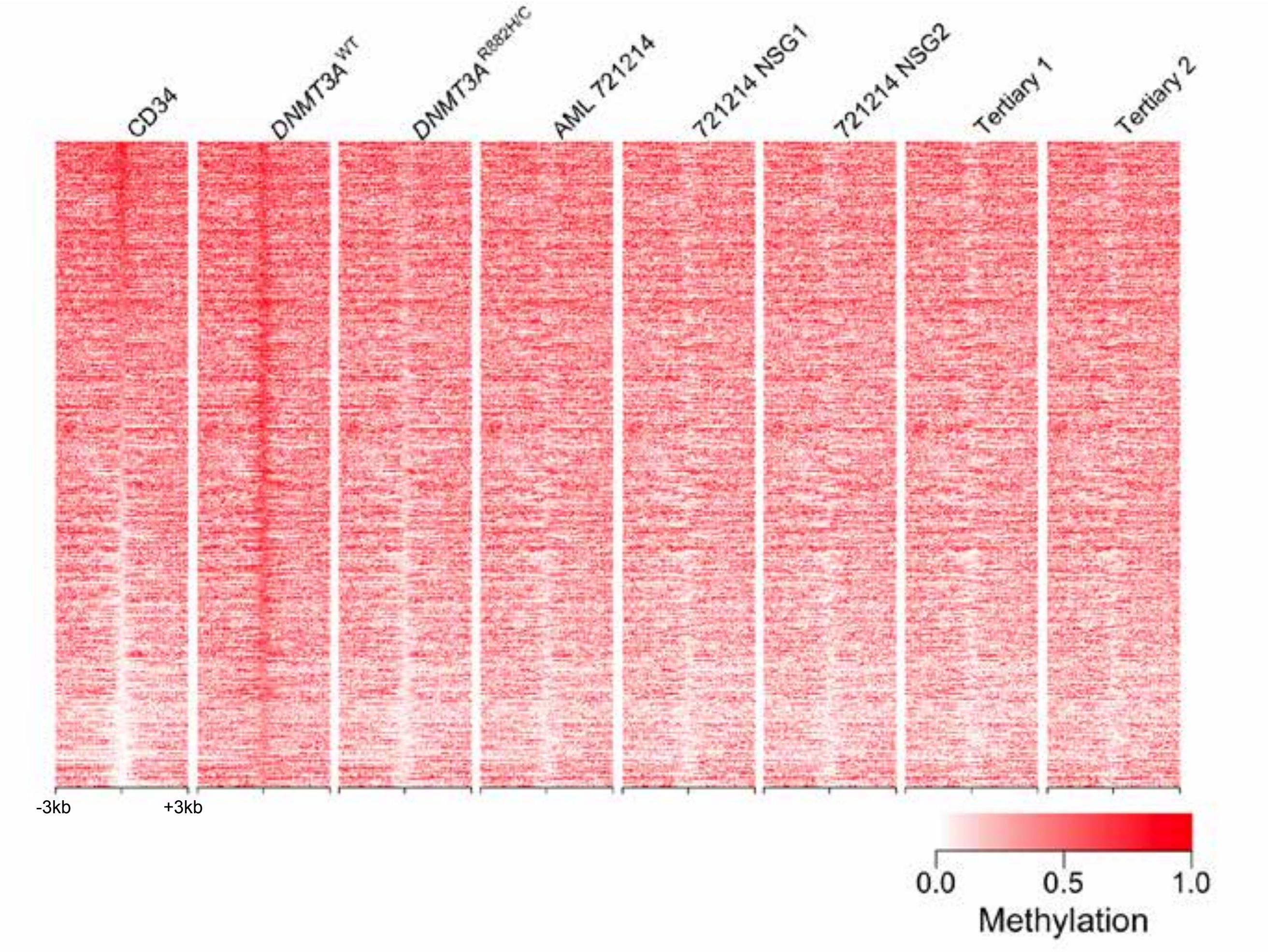

Figure S6. Expression DNA methylation genes in Kasumi-1, NB4, and OCI-AML3 cells.

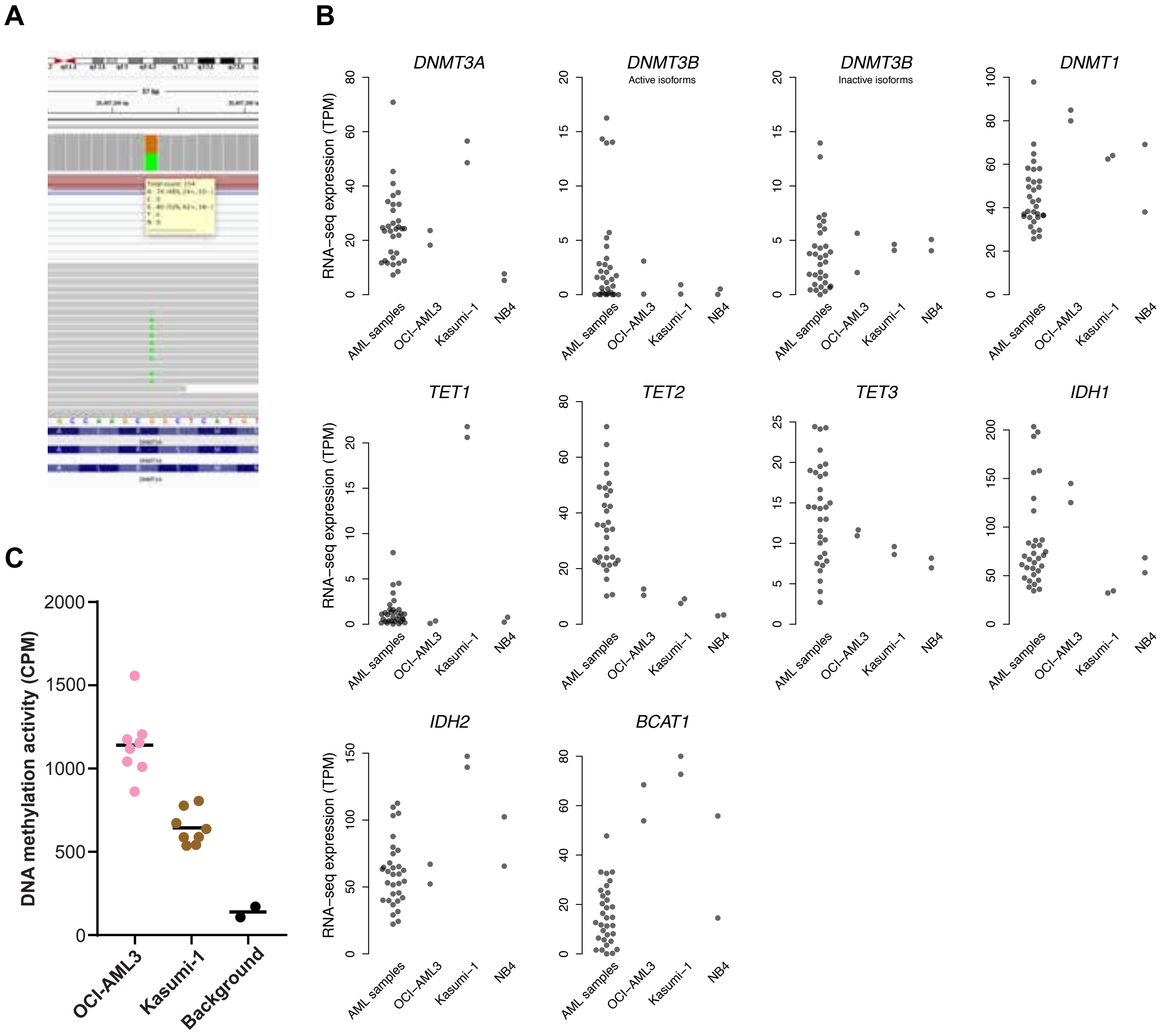

Supplement: Supplementary file 2 — Supplemental Figures [file 41408_2018_72_MOESM2_ESM.pdf]
